# A Hybrid Sequential Feature Selection Approach for Identifying New Potential mRNA Biomarkers for Usher Syndrome Using Machine Learning

**DOI:** 10.3390/biom15070963

**Published:** 2025-07-04

**Authors:** Rama Krishna Thelagathoti, Wesley A. Tom, Dinesh S. Chandel, Chao Jiang, Gary Krzyzanowski, Appolinaire Olou, M. Rohan Fernando

**Affiliations:** Molecular Diagnostic Research Laboratory, Center for Sensory Neuroscience, Boys Town National Research Hospital, Omaha, NE 68131, USA; ramakrishna.thelagathoti@boystown.org (R.K.T.); wesley.tom@boystown.org (W.A.T.); dinesh.chandel@boystown.org (D.S.C.); chao.jiang@boystown.org (C.J.); gary.krzyzanowski@boystown.org (G.K.); appolinaire.olou@boystown.org (A.O.)

**Keywords:** hybrid feature selection, machine learning, Usher syndrome, mRNA, biomarker detection, feature selection, transcriptomics, biomarker validation, genetic disorder detection

## Abstract

Usher syndrome, a rare genetic disorder causing both hearing and vision loss, presents significant diagnostic and therapeutic challenges due to its complex genetic basis. The identification of reliable biomarkers for early detection and intervention is crucial for improving patient outcomes. In this study, we present a machine learning-based hybrid sequential feature selection approach to identify key mRNA biomarkers associated with Usher syndrome. Beginning with a dataset of 42,334 mRNA features, our approach successfully reduced dimensionality and identified 58 top mRNA biomarkers that distinguish Usher syndrome from control samples. We employed a combination of feature selection techniques, including variance thresholding, recursive feature elimination, and Lasso regression, integrated within a nested cross-validation framework. The selected biomarkers were further validated using multiple machine learning models, including Logistic Regression, Random Forest, and Support Vector Machines, demonstrating robust classification performance. To assess the biological relevance of the computationally identified mRNA biomarkers, we experimentally validated candidates from the top 10 selected mRNAs using droplet digital PCR (ddPCR). The ddPCR results were consistent with expression patterns observed in the integrated transcriptomic metadata, reinforcing the credibility of our machine learning-driven biomarker discovery framework. Our findings highlight the potential of machine learning-driven biomarker discovery to enhance the detection of Usher syndrome.

## 1. Introduction

Usher syndrome (USH) is a rare genetic disorder that primarily causes sensorineural hearing loss, retinitis pigmentosa (RP), and, in some cases, vestibular dysfunction [1]. USH is a major contributor to inherited deaf-blindness and leads to severe impairments in those affected [2,3]. It is classified into four clinical subtypes (Usher types I, II, III, and IV) based on the severity and onset of symptoms [4,5]. Among these, Usher syndrome type I (USH1) is the most severe form, characterized by profound hearing loss and vestibular dysfunction present from birth. Usher syndrome type II (USH2) is associated with milder congenital hearing loss and normal vestibular function. Usher syndrome type III (USH3) is relatively rare. In USH3, hearing loss is progressive, leading to varying degrees of vestibular dysfunction [6,7]. Type IV (USH4), like type III, has progressive hearing loss and vision loss, but the vision impairment and balance issues tend to occur later in life [5]. The disorder is caused by mutations in several genes, including *MYO7A*, *CDH23*, *USH1C*, *USH2A*, *CLRN1*, and *ARSG*. which are crucial for the proper functioning of sensory hair cells in the inner ear and photoreceptor cells in the retina [5,8,9]. The genetic complexity and variable phenotypic presentation of Usher syndrome make it challenging to diagnose, especially in early stages when symptoms may be mild or nonspecific [10]. Traditional diagnostic approaches rely on clinical assessments and genetic testing, which, while informative, may not always provide conclusive results due to genetic heterogeneity and potential unknown causative mutations [11,12,13]. Thus, there is a pressing need for novel biomarkers that can facilitate the diagnosis of Usher syndrome.

Messenger RNA (mRNA) plays a fundamental role in gene expression by acting as the intermediary between DNA and protein synthesis [14,15]. Dysregulation of mRNA expression has been implicated in numerous genetic disorders, including Usher syndrome, where mutations in key genes lead to altered transcriptional profiles [16,17]. Recent studies have explored the role of mRNA in Usher syndrome and have identified specific expression patterns associated with disease progression [18]. These findings suggest that mRNA expression levels could serve as potential biomarkers for distinguishing Usher syndrome from unaffected individuals. Most previous work has focused on mRNA expression in the genes with variants causing Usher, particularly in tissues of the inner ear and retina. Retinal biopsy samples are very rare due to the risk of blindness, and inner ear hair cells almost exclusively come from cadavers. Induced pluripotent stem cells (iPSCs) can be used to study these cell types in vitro or as treatment [19,20,21,22]. However, iPSCs are not ideal for rapid diagnostics purposes as such. There remains a potential to uncover dysregulated mRNA non-invasive biospecimens which could serve as potential biomarkers for Usher syndrome. B-lymphocytes are readily available from a minimally invasive blood draw and have been shown to be useful in biomarker detection [23,24,25]. Additionally, B-lymphocytes can be immortalized easily using the Epstein–Barr virus (EBV) for future studies [26]. Thus, this study utilized machine learning characterization of mRNA expression profiles from immortalized B-lymphocytes from Usher syndrome patients and healthy controls in order to identify mRNA biomarkers for Usher syndrome.

A biomarker is a measurable biological molecule that serves as an indicator of a physiological or pathological process or a response to a therapeutic intervention [27,28]. The identification of reliable biomarkers is crucial for early diagnosis, monitoring disease progression, and evaluating treatment efficacy. In the context of Usher syndrome, mRNA biomarkers offer a promising avenue due to their direct involvement in gene expression and disease pathology [29]. For example, recent work has shown the utility of microRNA (miRNA) as a biomarker in the detection of USH [30,31]. Unlike DNA mutations, which are static and may not always correlate with disease severity, mRNA levels provide dynamic information about cellular changes and pathological progression. Leveraging mRNA biomarkers can thus improve diagnostic accuracy and facilitate personalized treatment strategies for individuals with Usher syndrome [31].

One of the primary challenges in identifying mRNA biomarkers is the high dimensionality of transcriptomic data [32,33]. A typical mRNA dataset consists of thousands of genes, many of which exhibit variations unrelated to the disease state. The presence of noise and irrelevant features complicates the identification of robust biomarkers [34,35]. Machine learning-based feature selection techniques provide an effective solution to this challenge by systematically narrowing down the most informative mRNAs associated with Usher syndrome [36,37]. By employing algorithms such as recursive feature elimination, LASSO regression, and mutual information-based selection, researchers can enhance the specificity and sensitivity of biomarker discovery [38].

In the field of feature selection, hybrid feature selection approaches have emerged as a powerful strategy for biomarker identification [39,40]. Hybrid feature selection is the process of combining multiple feature selection methods to achieve more robust and reliable results [41,42]. Unlike single-method approaches, which may be biased toward specific data properties, hybrid feature selection integrates various techniques to leverage their complementary strengths. This method enhances the stability and reproducibility of selected biomarkers, ensuring that the identified mRNAs are truly relevant for Usher syndrome classification [43,44,45]. In this study, we analyzed high-dimensional mRNA data from Usher syndrome samples and applied a hybrid feature selection approach to identify potential mRNA biomarkers capable of distinguishing Usher from control samples. The implementation of these methods aims to support the early and accurate identification of Usher syndrome, allowing for the timely initiation of clinical management and personalized intervention strategies.

## 2. Related Work

The use of machine learning and hybrid feature selection methods has become increasingly prominent in the analysis of high-dimensional RNA seq data for disease diagnosis. In the context of Usher syndrome, Thelagathoti et al. (2025) applied ensemble feature selection integrated with nested cross-validation for identifying miRNA-based biomarkers, establishing a strong precedent for the computational detection of this rare genetic disorder [46]. However, mRNA signatures offering more direct gene-level insights remain underexplored for this condition. Prior studies such as Yousef et al. (2021) introduced miRcorrNet, a feature grouping and ranking framework integrating miRNA and mRNA profiles, but without the dedicated modeling of mRNA features or thorough experimental validation [47]. Similarly, Chinnaswamy and Srinivasan (2015) utilized correlation-based hybrid feature selection with particle swarm optimization on microarray data, though their work lacked multi-step refinement or nested validation to ensure generalizability [48]. More recent work by Han et al. (2022) used machine learning and WGCNA to identify mRNA markers in ankylosing spondylitis, and Kong et al. (2025) combined hybrid feature extraction with ensemble learning to classify mRNA localization, but neither study focused on rare syndromic disorders or linked findings to diagnostic validation [49,50].

Complementary studies have shown the effectiveness of recursive ensemble feature selection (REFS) in generating robust mRNA-based disease signatures. Metselaar et al. (2021) demonstrated REFS as a reliable strategy for deriving predictive mRNA signatures in chronic fatigue syndrome [51], while Kidwai et al. (2023) employed a similar pipeline to predict treatment responsiveness in moderate-to-severe asthma patients using transcriptomic profiles [52]. These studies emphasize the strength of combining recursive selection and ensemble models in enhancing model robustness. However, they remain specific to immune-related conditions and do not validate their findings through experimental platforms such as ddPCR. Our current study builds on these advances by implementing a hybrid sequential feature selection strategy within a nested cross-validation design. Unlike prior works, we target Usher syndrome, a rare genetic disorder, and validate our top-ranked mRNA biomarkers using droplet digital PCR, bridging the gap between computational predictions and biological validation. This integrative pipeline contributes a novel, reproducible, and biologically grounded framework for mRNA biomarker discovery in the context of rare disease diagnostics.

## 3. Materials and Methods

### 3.1. Experimental Design

Three of the cell lines used in this study, USH1B, USH1D, and USH3A were developed in Dr. William J. Kimberling’s lab at Boys Town National Research Hospital (Omaha, NE, USA) by immortalizing lymphocytes from Usher syndrome patients with Epstein–Barr virus (EBV, B95-8 strain). Informed consent was obtained, and protocols were approved by the Boys Town IRB (IRB#96-06-0X). The patients’ ages at diagnosis are unknown, but blood was drawn shortly after diagnosis. One 36-year-old male, and one 45-year-old male healthy donor’s lymphocytes were similarly transformed to serve as a control. Additionally, a USH2A B-cell line (GM09053) from a 9-year-old patient was sourced from the Coriell Institute (Camden, NJ, USA). All lines were cultured in RPMI 1640 with 20% FBS and 50 µg/mL gentamicin, maintained at 37 °C in 5% CO_2_ using 100 nm × 20 mm tissue culture plates. All mRNA for the analysis is derived from the above B-lymphocyte cell lines. RNA for each Usher cell line was extracted in triplicate, and the two healthy control cell lines were extracted in quadruplicate for mRNA library preparation and subsequent next generation sequencing (NGS).

### 3.2. RNA Sequencing and Processing

Messenger RNA was extracted from patient derived B-lymphocyte cell lines described previously [30]. Briefly, total RNA was extracted from four B-lymphocyte cell lines representing Usher subtypes USH1B, USH1D, USH2A, and USH3A, using GeneJET™ RNA Purification Kit (cat. # K0731; Thermo Fisher Scientific, Waltham, MA, USA) following the manufacturer’s recommend protocol. Each line had four technical replicates for a total of 16 USH RNA samples. Additionally, four B-lymphocyte control cell lines were also processed in quadruplicate for this analysis. In total, 32 mRNA-seq libraries were sequenced on the Illumina NovaSeq platform (150bp paired-end reads) to an average read depth of 50.17 million reads per sample (Illumina Inc., San Diego, CA, USA). However, of the original 32 samples, 4 samples were untransformed and thus excluded from the analysis. Raw fastq reads underwent adapter trimming and low-quality bases were removed using the BBMap suite’s BBDuk function (Bushnell, Brian. “BBMap: a fast, accurate, splice-aware aligner.” (2014)). Trimmed reads were then aligned to GENCODE human genome transcripts (release 47 GRCh38.p14) using STAR aligner [53]. Transcript quantification was conducted using Salmon [54]. Transcript quantities from Salmon were used for all machine learning methodologies described below.

### 3.3. Overview of Machine Learning Pipeline

**Figure 1:**
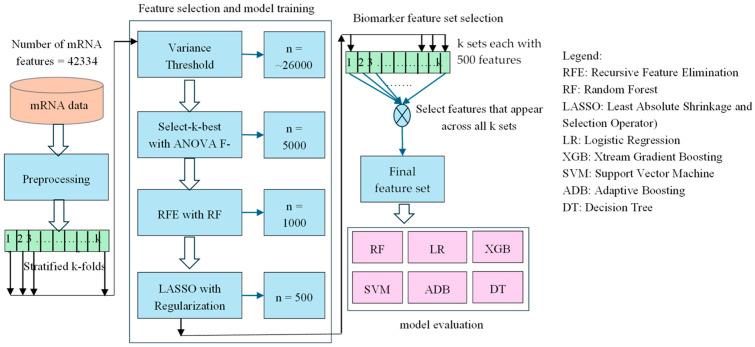
A hybrid sequential feature selection pipeline combining Variance Threshold, ANOVA F and LASSO to identify robust features, followed by classification using six machine learning models. The final feature set is derived from features commonly selected across all stages.

The overall methodology employed in this study shown in Figure 1 involves a machine learning pipeline designed to identify a robust biomarker set from high-dimensional mRNA expression data for Usher syndrome classification. The pipeline integrates data preprocessing, feature selection, and classification to enhance predictive accuracy while minimizing noise and overfitting. Given the complexity of mRNA datasets, a systematic feature selection approach is essential to extract biologically relevant information while ensuring model interpretability and stability. To generate our results, we have used a fixed random seed of 42 across all code blocks to ensure reproducibility of our experiments. Additionally, we have updated the manuscript to reflect this. The software environment used for analysis includes Python version 3.12.6 and JupyterLab version 4.2.5.

### 3.4. Preprocessing and Cross-Validation

The dataset consists of mRNA expression profiles from 28 samples, comprising Usher syndrome and control samples. Each sample includes 42,334 mRNA features. First, the raw mRNA expression data is normalized to ensure consistency across samples and mitigate batch effects. Normalization is crucial for maintaining uniformity in expression levels, thereby improving the reliability of downstream analyses. Then the dataset is split using stratified 5-fold cross-validation to maintain class distribution across training and validation sets. This ensures that the model is trained and validated on representative samples, preventing bias due to class imbalance [55].

### 3.5. Hybrid Feature Selection Approaches

The selection of informative features from high-dimensional mRNA expression data is crucial for improving classification performance, reducing computational complexity, and mitigating the risk of overfitting. In this study, a hybrid feature selection approach is employed to systematically refine the initial feature space of 42,334 mRNA features. This multi-stage strategy integrates statistical filtering, tree-based ranking, and regularization techniques to ensure the selection of biologically relevant and discriminatory features. The rationale for this approach is to balance the strengths of different methodologies, thereby capturing essential signal variations while eliminating redundant or noisy features. By leveraging multiple selection criteria, the final feature set is optimized for robust and generalizable classification performance.

#### 3.5.1. Variance Threshold: Initial Filtering

The first stage of feature selection employs a variance threshold method to eliminate features with minimal variation across samples. Features with near-constant expression values provide little to no discriminative power and can introduce noise into the model. By setting a threshold variance of 0.01, only those features exhibiting sufficient variability are retained [56]. This preprocessing step significantly reduces the dimensionality of the dataset while preserving features with potential biological significance.

The variance of each feature was computed using the following equation:

(1) $${Variance}_{j}=\frac{1}{n}\sum_{i=1}^{n}{\left(x_{ij}-\bar{x_{j}}\right)}^{2}$$

where

- $x_{ij}$ is the value of feature j for sample i
- $x_{j}$ is the mean value of feature j

This implies variance thresholding will retain the feature j only if ${Variance}_{j}>0.01$.

#### 3.5.2. Univariate Feature Selection: ANOVA F-Test

Following the initial filtering, univariate feature selection is performed using an Analysis of Variance (ANOVA) F-test. This statistical method evaluates the relationship between each feature and the target class labels, selecting the top 5000 features based on their F-scores [56]. This statistical method evaluates whether the meaning of a feature differs significantly between classes. The F-score is computed using the following formula:

(2) $$F=\frac{Variance\;between\;groups}{Variance\;within\;groups}$$

Features with high F-scores are retained, suggesting strong class separation. The ANOVA F-test is particularly effective in identifying features that exhibit significant differential expression between classes, thereby ensuring that the retained features contribute to the classification task. This step enhances the interpretability of the selected features by prioritizing those with the highest statistical relevance.

#### 3.5.3. Recursive Feature Elimination with Random Forest

To further refine the feature set, Recursive Feature Elimination (RFE) is applied using a Random Forest classifier. This method ranks features based on their importance scores derived from a trained ensemble of decision trees [57]. The top 1000 most influential features are retained for the subsequent stage. Unlike univariate statistical tests, Random Forest inherently captures complex, non-linear relationships between features and class labels, making it a robust technique for feature selection. Additionally, the ability of Random Forest to handle feature interactions enhances the stability and reliability of the selected subset. Random Forest assigns importance based on how much a feature improves classification at decision splits:

(3) $${Importance}_{j}=\sum \left(Decrease\;in\;impurity\;using\;feature\;j\right)$$

We remove the least important features step-by-step and keep the top 1000 based on these scores.

#### 3.5.4. LASSO Regularization: Final Feature Selection

In the final stage, L1-regularized regression, also known as Least Absolute Shrinkage and Selection Operator (LASSO), is employed to select the 500 most predictive features. LASSO performs feature selection by penalizing less important features, forcing their regression coefficients to zero [57]. This is achieved by minimizing the size of regression coefficients computed using the following formula:

(4) $$\sum {\left(y_{i}-\hat{y_{i}}\right)}^{2}+\lambda \sum |\beta_{j}|\;\;\;\;\;\;\;$$

As λ increases, more βj are shrunk to zero, effectively removing non-informative features. This method effectively eliminates multicollinear features while retaining only the most influential predictors. The use of LASSO ensures sparsity in the final model, thereby improving interpretability and reducing overfitting.

### 3.6. Classification Models

The final biomarker set obtained from the hybrid feature selection approach is used as input for multiple classification algorithms to evaluate predictive performance. The classifiers employed include Logistic Regression, Random Forest, XGBoost, AdaBoost, Decision Tree, Support Vector Machine (SVM), and Naïve Bayes [58,59].

#### 3.6.1. Logistic Regression

Logistic regression is a foundational statistical method used for binary classification tasks. It models the relationship between a set of input features and the probability of a particular class label by applying a logistic (sigmoid) transformation to a linear combination of the input variables [60]. Despite its simplicity, logistic regression performs well when the classes are linearly separable and the data is well-behaved. In biomedical applications, it is often used due to its interpretability and the ability to quantify feature contributions to disease risk or condition presence. Logistic function is given as follows.

(5) $$P\left(\;y=\frac{1}{x}\right)=\frac{1}{1+e^{-(\beta 0+\sum_{i=1}^{n}\beta_{i}x_{i})}}$$

where $\beta_{i}$ are coefficients [60]. This equation models the probability that a sample belongs to a class using the logistic function.

#### 3.6.2. Random Forest

Random Forest is an ensemble learning method that builds multiple decision trees during training and outputs the class that is the majority vote among the individual trees [61]. Each tree is trained on a bootstrap sample of the data, and feature selection for splits is randomized, which adds diversity and reduces overfitting. Random Forest is particularly robust against noise and capable of handling non-linear relationships and high-dimensional data. In healthcare and genomics, it is commonly used due to its high accuracy, built-in feature importance estimates, and minimal parameter tuning. Importance for each feature is determined by using the following formula

(6) $$Gini=\sum_{i=1}^{n}{p_{i}}^{2}$$

where *i* is the number of classes and $p_{i}$ is the proportion of samples belonging to class *i.*

#### 3.6.3. XGBoost (EXtream Gradient Boosting)

XGBoost is an advanced implementation of gradient boosting machines that has gained popularity for its high efficiency and performance on structured data. It builds trees sequentially, where each new tree corrects the errors of the previous ensemble using gradient descent optimization on a custom loss function [62]. XGBoost supports regularization, which controls overfitting and enhances model generalization. It is particularly suited for imbalanced datasets and is widely used in Kaggle competitions and biomedical research alike. The following is the objective function of XGBoost.

(7) Total Loss=Sum of (Prediction Error for each data point)+Regularization Term

Each round of training adds a new model to minimize the overall prediction error. The regularization term prevents overly complex models by penalizing model size and weight.

#### 3.6.4. Support Vector Machine

Support Vector Machine (SVM) is a powerful classification algorithm that identifies the optimal hyperplane that best separates the data into distinct classes. It maximizes the margin between the nearest points of different classes, known as support vectors [63]. SVM is particularly effective in high-dimensional spaces and can handle non-linear boundaries through kernel functions (e.g., radial basis function). It is widely used in bioinformatics for gene expression classification, disease prediction, and biomarker discovery due to its robustness in complex datasets. The model tries to draw a line (or hyperplane) that maximally separates the classes while misclassifying as few points as possible.

In simple terms:

Maximize Margin while ensuring: 

Class_Label×(Weight_Vector · Feature_Vector+Bias) ≥ 1

#### 3.6.5. AdaBoost

AdaBoost is a boosting technique that combines multiple weak learners, typically decision stumps, into a strong classifier by iteratively focusing more on the misclassified samples. During each iteration, sample weights are adjusted so that subsequent models pay more attention to difficult examples [64]. AdaBoost is sensitive to noisy data but can achieve high accuracy on clean datasets with simple base learners. In clinical studies, AdaBoost has been used for risk prediction and biomarker identification due to its model interpretability and adaptive nature. AdaBoost

(8) $$Final\;Prediction=Sum\;\left(Alpha_{t}\times Weak_{Learner_{t_{Prediction}}}\right)$$

$${Alpha}_{t}=0.5\times log((1-Error\_t)/Error\_t)$$

Alpha determines how much influence each weak learner has. Learners with lower error achieved higher weight in the final prediction.

#### 3.6.6. Decision Tree

Decision Trees classify data by recursively partitioning it into subsets based on feature values. At each node, the algorithm selects the feature and threshold that best separates the classes, typically using impurity measures like the Gini index (as shown in Equation (6)) or entropy [65]. Decision trees are intuitive and easy to visualize, making them useful for explaining decision-making processes. However, they are prone to overfitting, which can be mitigated by pruning or using ensemble approaches like Random Forest and Gradient Boosting.

#### 3.6.7. Naïve Bayes

Naïve Bayes is a probabilistic classifier based on Bayes’ theorem, assuming independence among features [66]. Despite this strong and often unrealistic assumption, Naïve Bayes performs remarkably well in various real-world scenarios, particularly in text classification and spam detection. In biomedical applications, it offers a fast and scalable approach to disease classification, especially when dealing with large-scale, sparse data. Its simplicity and interpretability make it a preferred choice for initial model benchmarking.

(9) $$P\left(A/B\right)=\frac{P(B/A)*(P(A))}{P(B)}$$

where P(A/B) is the probability of event A when B occurs, P(A) is the probability event A will occur, P(B/A) is the probability event B will occur given A occurs, and P(B) is the probability event B will occur.

### 3.7. Selection of Robust Features Across Cross-Validation Folds

To ensure stability and generalizability, the final feature set comprises features that consistently appear across all five cross-validation folds. This consistency check minimizes the risk of dataset-specific bias and enhances the reliability of the selected biomarkers. By integrating multiple selection strategies and validating feature stability, this hybrid approach provides a robust and systematic framework for identifying biologically meaningful features in high-dimensional mRNA datasets.

This multi-stage feature selection process enhances the interpretability, reliability, and predictive performance of machine learning models in the context of Usher syndrome classification. The integration of statistical, ensemble-based, and regularization techniques ensures that the selected features are both relevant and generalizable for downstream analysis.

## 4. Results

### 4.1. Identified mRNA Biomarkers

**Figure 2:**
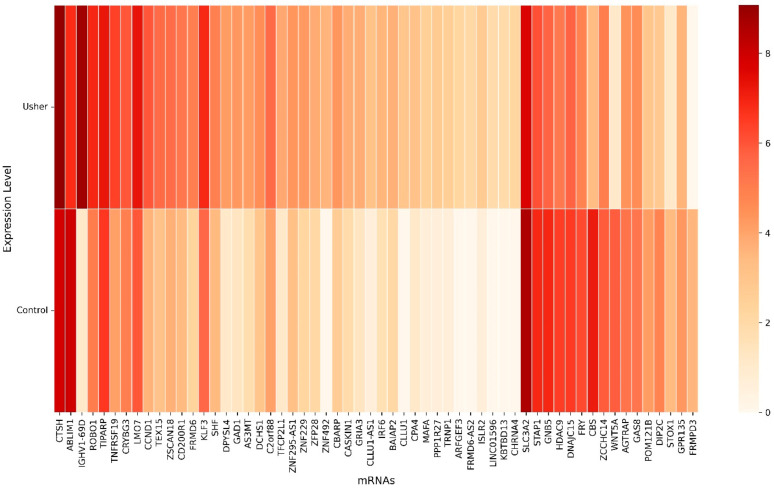
The heatmap shows 58 selected key mRNAs.

Using a machine learning-based hybrid sequential feature selection approach, we identified 58 key mRNA biomarkers associated with Usher syndrome (Figure 2). The heatmap illustrates the expression levels of these selected genes, where rows represent individual mRNA biomarkers and columns denote conditions (Control and Usher). The color gradient ranges from light (low expression) to dark (high expression), indicating differential gene expression between the two conditions. Several genes, such as *IGHV1-69D, CTSH*, and *LMO7*, exhibited significant upregulation in Usher syndrome, while others, including *SLC3A2, ABLIM1*, and *CBS*, were notably downregulated.

**Figure 3:**
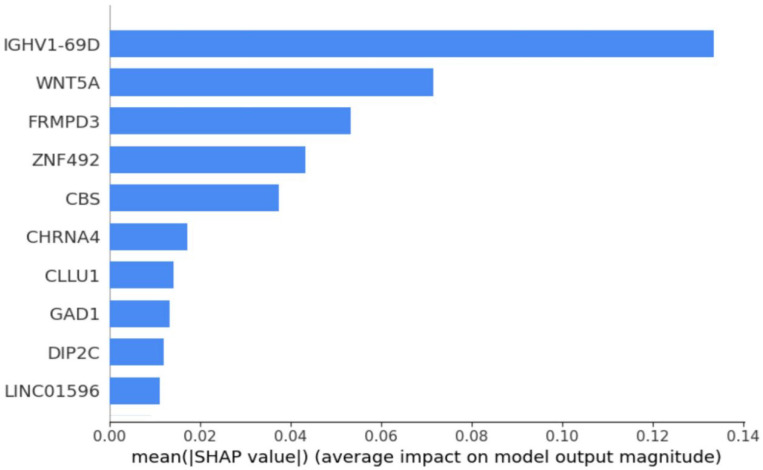
SHAP feature importance plot that shows top 10 mRNAs.

Further analysis using SHAP feature importance plot [67] in Figure 3 highlights the top 10 most discriminative mRNAs: *IGHV1-69D, WNT5A, FRMPD3, ZNF492, CBS, CHRNA4, CLLU1, GAD1, DIP2C,* and *LINC01596*. Among these, *WNT5A* and *CBS* were significantly downregulated in Usher syndrome, whereas the remaining eight genes showed upregulation. Notably, *IGHV1-69D* emerged as the most distinctive biomarker, underscoring its potential for differentiating Usher syndrome from control samples. These findings suggest that mRNAs such as *WNT5A* and *IGHV1-69D* could serve as robust biomarkers for Usher syndrome detection.

### 4.2. Validation of mRNA Biomarkers Using Droplet Digital-PCR (ddPCR)

**Figure 4:**
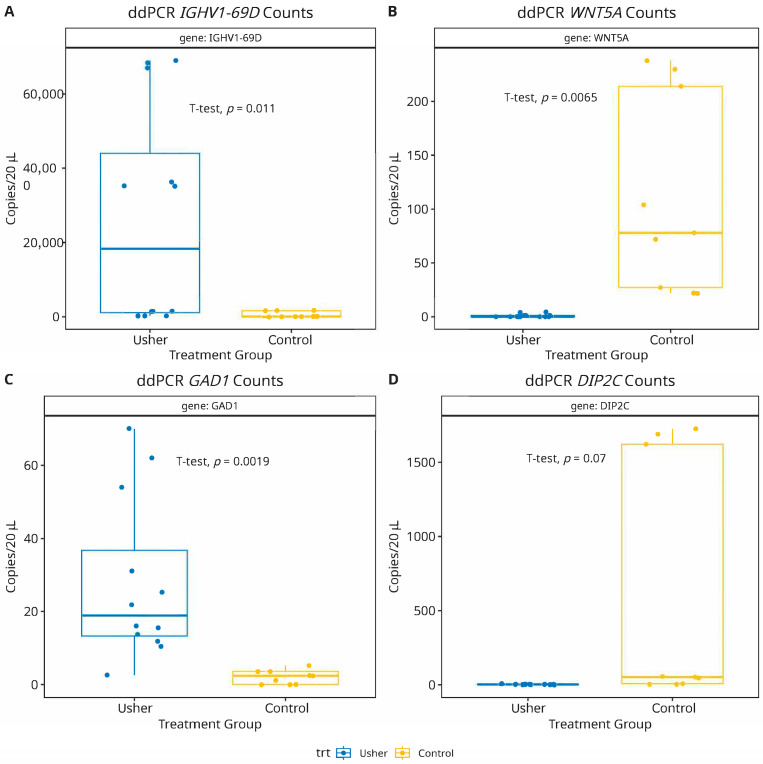
Droplet digital-PCR (ddPCR) assay validation of mRNA biomarkers shows results consistent with computational predictions. Two mRNAs: IGHV1-69D (panel-**A**) and GAD1 (panel-**C**) were significantly (*p* = 0.01; 0.001, respectively) upregulated, while WNT5A (panel-**B**) showed a significant (*p* = 0.006) down regulation in Usher samples. The mRNA DIP2C also depicted a non-significant (*p* = 0.07) reduced expression in Usher, compared to healthy controls (panel-**D**).

To validate the predictive accuracy and biological relevance of the machine learning-identified mRNA biomarkers, we selected four mRNAs representing both upregulated and downregulated genes from the top 10 ranked candidates (Figure 3). According to our meta-analysis of transcriptomic data, IGHV1-69D and GAD1 are significantly upregulated, whereas WNT5A and DIP2C are significantly downregulated in Usher syndrome. To experimentally verify these predictions, we performed droplet digital-PCR (ddPCR) assays on the selected mRNA panel to compare their absolute counts due to differentially expressed mRNA-profiles in Usher samples [68,69]. As shown in Figure 4, the ddPCR results were consistent with our computational predictions. IGHV1-69D and GAD1 demonstrated statistically significant upregulation in Usher syndrome samples compared to controls (*p* < 0.05), while WNT5A and DIP2C exhibited a significant downregulation (*p* < 0.05). The successful validation of these markers not only supports their potential involvement in the disease’s molecular etiology but also highlights the effectiveness of our approach in translating high-dimensional transcriptomic signals into biologically meaningful candidates for future diagnostic or therapeutic exploration.

### 4.3. Model Training and Validation

Initially, a stratified cross-validation (CV) approach, combined with hybrid feature selection approaches (detailed in Section 3) was utilized to select the key mRNAs. Specifically, we used stratified k-fold CV to divide the dataset into multiple folds while preserving the proportion of Usher syndrome and control samples within each fold. In each split, one fold was used for validation while the remaining folds were used for model training. In this process, each CV fold produces 500 mRNAs. At the end of the process, key mRNAs that appear consistently across all CV folds were chosen as biomarker mRNAs. Simultaneously, seven machine learning algorithms were also trained and validated across each CV fold. The average performance of each model was evaluated using key metrics including accuracy, sensitivity, specificity, F1 score, and Area Under the Curve (AUC). These metrics are computed using standard classification metrics derived from the confusion matrix, which are described below in Table 1. The average performance metrics are shown in Table 2.

Where

- TP = True Positives (real positives predicted as positives);
- FN = False Negatives (real positives incorrectly predicted as negatives);
- FP = False Positives (real negatives incorrectly predicted as positives);
- TN = True Negatives (real negatives correctly predicted as negatives).

Performance Metric Formulas

1. Accuracy

$$Accuracy=\frac{\left(TP+TN\right)}{\left(TP+TN+FP+FN\right)}$$

2. Sensitivity (Recall or True positive rate)

$$Sensitivity=\frac{TP}{\left(TP+FP\right)}$$

3. Specificity

$$Specificity=\frac{TN}{\left(TN+FP\right)}$$

4. F1 score

$$F1\;score=\frac{2\times (Precision\times Recall)}{\left(Precision+Recall\right)}$$

where $Precision=\frac{TP}{\left(TP+FP\right)}$.

5. Area Under the Curve (AUC)

Calculated from the ROC curve plotting True Positive Rate (Sensitivity) against False Positive Rate (FPR), where

$$FPR=\frac{FP}{\left(FP+TN\right)}$$

**Table 2:** Model training performance.

Model	Average Accuracy	Average Sensitivity	Average Specificity	Average F1 Score	Average AUC
Logistic Regression	0.9667 ± 0.07 (0.90, 1.00)	1.0000 ± 0.00 (1.00, 1.00)	0.9333 ± 0.15 (0.89, 1.00)	0.9714 ± 0.06 (0.92, 1.00)	0.9444 ± 0.12 (0.87, 1.00)
Random Forest	**0.9667 ± 0.07 (0.93, 1.00)**	**1.0000 ± 0.00** **(1.00, 1.00)**	**0.9333 ± 0.15** **(0.90, 1.00)**	**0.9714 ± 0.06** **(0.94, 1.00)**	**1.0000 ± 0.14 (0.90, 1.00)**
XGBoost	0.8667 ± 0.14 (0.74, 0.99)	0.9500 ± 0.11 (0.85, 1.00)	0.7667 ± 0.22 (0.57, 0.96)	0.8929 ± 0.11 (0.80, 0.99)	0.8583 ± 0.15 (0.72, 0.99)
AdaBoost	0.8667 ± 0.14 (0.67, 0.91)	0.9333 ± 0.28 (0.51, 0.99)	0.7667 ± 0.24 (0.63, 1.00)	0.8878 ± 0.18 (0.62, 0.94)	0.8500 ± 0.15 (0.66, 0.92)
Decision Tree	0.9000 ± 0.17 (0.85, 0.98)	0.9333 ± 0.29 (0.91, 1.00)	0.8333 ± 0.24 (0.80, 1.00)	0.9092 ± 0.21 (0.84, 1.00)	0.8833 ± 0.18 (0.83, 0.98)
SVM	0.8333 ± 0.17 (0.69, 0.98)	1.0000 ± 0.00 (1.00, 1.00)	0.6333 ± 0.34 (0.33, 0.93)	0.8778 ± 0.13 (0.77, 0.99)	0.8889 ± 0.19 (0.72, 1.00)
Naive Bayes	0.9000 ± 0.09 (0.82, 0.98)	1.0000 ± 0.00 (1.00, 1.00)	0.7667 ± 0.22 (0.57, 0.96)	0.9206 ± 0.07 (0.86, 0.99)	0.8833 ± 0.11 (0.79, 0.98)

Best performing model is in bold text.

Selected biomarker mRNAs were further utilized to validate using seven machine learning models: Logistic Regression, Random Forest, XGBoost, AdaBoost, Decision Tree, Support Vector Machine (SVM), and Naïve Bayes. We used the default hyperparameters for all machine learning models as provided by their respective libraries. No systematic hyperparameter tuning (e.g., grid search or random search) was performed in the current implementation. The validation results are as shown in Table 3.

The results from the model training and feature selection phase indicate that most machine learning models achieved high performance metrics, with Logistic Regression and Random Forest models showing accuracy levels of 96.67%. However, among the models evaluated, XGBoost has the highest accuracy (96.67%), perfect sensitivity (100%), and strong specificity (93.33%), along with an F1 score of 0.9714 and an AUC of 0.9667. Decision Tree performed well with an accuracy of 96.67%, sensitivity of 95%, and perfect specificity (100%), resulting in an F1 score of 0.9714 and AUC of 0.9750. While its specificity was excellent, it had slightly lower sensitivity compared to XGBoost, making it less effective in identifying all true positives. Random Forest achieved perfect sensitivity (100%) but had an accuracy of 90% and specificity of 80%. Its F1 score of 0.9214 and AUC of 0.9778 indicate robustness, though it produced more false positives than Decision Tree and XGBoost. Logistic Regression, with 86.67% accuracy, 90% sensitivity, and 86.67% specificity, performed decently but lagged behind the top three models in sensitivity and specificity. Its F1 score of 0.8833 and AUC of 0.9333 show a reasonable balance, though less effective overall. AdaBoost, with an accuracy of 88.67%, sensitivity of 93.33%, and specificity of 80%, was less effective than the top models due to lower specificity and AUC of 0.8667, despite performing reasonably well overall. SVM performed poorly with 73.33% accuracy, 80% sensitivity, and 66.67% specificity, leading to a low F1 score of 0.75 and AUC of 0.5778, making it the least effective model. On the other hand, Naive Bayes delivered perfect results across all metrics (100% accuracy, sensitivity, specificity, F1 score, and AUC of 1.0) but may suffer from overfitting and assumptions of feature independence, limiting its real-world applicability.

In summary, XGBoost and Decision Tree were the best performers, offering a strong balance of sensitivity, specificity, and overall performance. Random Forest also performed well but had slightly lower specificity. Notably, both models exhibited low standard deviation (±0.07 for XGBoost and ±0.12 for Decision Tree in accuracy) and narrow confidence intervals (CI: 0.90–1.00 and 0.73–0.94, respectively), indicating stable and reliable predictions across validation folds. Logistic Regression and AdaBoost were outperformed by the top models, while SVM’s poor performance indicated it was not suitable for this dataset. Naive Bayes, despite perfect results, has limitations due to overfitting and feature assumptions.

## 5. Discussion

In this study, we employed a hybrid sequential feature selection approach integrated with machine learning to identify key mRNA biomarkers for Usher syndrome. In contrast, traditional differential expression analysis (DEA) methods, such as limma [70] and DESeq2 [71], use traditional generalized linear models (GLMs) to make inferences about differential abundance between case and control groups. These methods are widely used due to their interpretability and ability to detect significant expression changes; however, they may overlook subtle but biologically relevant interactions among genes and often require stringent *p*-value corrections to control false discoveries. The hybrid machine learning approach used in this study offers several advantages over DEA, including the ability to capture non-linear relationships, interactions among features, and complex expression patterns that may not be evident through traditional statistical methods. Moreover, by integrating multiple feature selection techniques, this approach enhances the robustness and reproducibility of biomarker discovery, making it a powerful tool for detection and therapeutic targets for Usher syndrome.

Additionally, the experimental validation of selected mRNA biomarkers using droplet digital PCR (ddPCR) adds a critical layer of biological relevance to the computational findings. The ddPCR results corroborated the expression trends observed in the transcriptomic dataset, reinforcing the reliability of the feature selection framework and the clinical potential of the identified markers. This alignment between computational prediction and wet-lab validation highlights the utility of hybrid machine learning pipelines not just in feature prioritization but also in driving biologically meaningful discoveries. Going forward, these validated biomarkers can be further explored in larger, multi-center cohorts to evaluate their utility in early diagnosis, patient stratification, and treatment monitoring for Usher syndrome.

Due to the rarity of Usher syndrome and the challenges associated with collecting high-quality patient samples, the available sample size for this study is inherently limited. Usher syndrome is a genetically heterogeneous and clinically complex disorder, making cohort assembly and standardized data collection particularly difficult. Despite these limitations, this study represents the first known attempt to apply machine learning methodologies to mRNA expression data specific to Usher syndrome. The hybrid sequential feature selection pipeline we employed identified a panel of 58 mRNAs with strong discriminatory power between Usher patients and controls. These mRNAs may serve as potential biomarkers for diagnostic or prognostic testing. Given their predictive relevance and biological plausibility, this curated set of transcripts offers a promising foundation for future validation studies and could be incorporated into molecular diagnostic assays to enhance early detection and classification of Usher syndrome.

Pathway analysis was performed using gprofiler to identify significantly enriched biological pathways associated with the provided gene list. This analysis reveals key biological mechanisms underlying the conditions studied. The identification of pathways related to otic development, neurotransmission, epigenetic regulation, and metabolic processes points to potential biomarkers and therapeutic targets [72,73,74,75,76,77,78,79,80,81,82]. Future research should focus on the functional validation of these pathways to better understand their precise roles in disease pathogenesis. A comprehensive list of enriched pathways, gene sets, and statistical outputs is provided in the Supplementary Materials for further reference.

## 6. Limitations and Future Directions

Despite the promising results of our machine learning-based hybrid sequential feature selection approach for mRNA biomarker discovery in Usher syndrome, several limitations should be acknowledged. The small sample size in our study may impact the generalizability of the identified biomarkers, necessitating larger datasets for improved robustness. Additionally, the biological significance of the selected mRNA features requires further experimental validation through in vitro and in vivo studies. To address these limitations, future studies should focus on expanding the dataset size and incorporating diverse populations to enhance the reliability of findings. Integrating multi-omics data, including proteomics, metabolomics, and epigenomics, could provide a more comprehensive understanding of Usher syndrome pathophysiology. Additionally, validating the selected biomarkers in independent datasets and prospective clinical studies will be crucial for assessing their diagnostic and prognostic relevance.

## 7. Conclusions

This study presents a machine learning-based hybrid sequential feature selection approach for identifying mRNA biomarkers associated with Usher syndrome. This study makes several notable contributions to the field of biomarker discovery in rare genetic disorders. First, we present a hybrid sequential feature selection framework that systematically combines multiple statistical and machine learning-based techniques—variance thresholding, ANOVA F-test, recursive feature elimination with Random Forest, and LASSO regression—to reduce dimensionality and identify highly informative mRNA biomarkers. Second, by employing a nested cross-validation strategy, we ensure rigorous and unbiased performance evaluation, addressing a key limitation in studies with small sample sizes. Third, we validate a subset of the top biomarkers using droplet digital PCR (ddPCR), providing experimental confirmation of computational predictions and reinforcing the translational value of our pipeline. Finally, this is one of the few studies to focus specifically on mRNA signatures for Usher syndrome, advancing the potential for early detection and precision diagnostics in this understudied rare disease. Together, these contributions demonstrate the strength of our data-driven, biologically informed approach and set the stage for future clinical and translational research. Despite these promising results, further validation on larger and independent cohorts is necessary to confirm the clinical relevance of the selected biomarkers.

## Figures and Tables

**Table 1:** Confusion matrix.

	Predicted Positive	Predicted Negative
**Actual Positive**	TP	FN
**Actual Negative**	FP	TN

**Table 3:** Model validation using selected features (Mean ± Std with 95% Confidence Interval).

Model	Accuracy	Sensitivity	Specificity	F1 Score	AUC
Logistic Regression	0.8667 ± 0.18 (0.71, 1.00)	0.9000 ± 0.22 (0.70, 1.00)	0.8667 ± 0.30 (0.61, 1.00)	0.8833 ± 0.16 (0.74, 1.00)	0.9333 ± 0.15 (0.80, 1.00)
Random Forest	0.9000 ± 0.15 (0.77, 1.00)	1.0000 ± 0.00 (1.00, 1.00)	0.8000 ± 0.30 (0.54, 1.00)	0.9214 ± 0.11 ](0.82, 1.00)	0.9778 ± 01.d0 (0.87, 1.00)
XGBoost	**0.9667 ± 0.07** **(0.90, 1.00)**	**1.0000 ± 0.00** **(1.00, 1.00)**	**0.9333 ± 0.15 (0.85, 1.00)**	**0.9714 ± 0.06** **(0.92, 1.00)**	**0.9667 ± 0.07 (0.90, 1.00)**
AdaBoost	0.8867 ± 0.14 (0.74, 0.99)	0.9333 ± 0.00 (1.00, 1.00)	0.8000 ± 0.30 (0.44, 0.96)	0.9111 ± 0.11 (0.81, 0.99)	0.8667 ± 0.15 (0.72, 0.98)
Decision Tree	0.9667 ± 0.12 (0.93, 0.94)	0.9500 ± 0.30 (0.94, 1.00)	1.0000 ± 0.24 (0.93, 1.00)	0.9714 ± 0.19 (0.94, 0.97)	0.9750 ± 0.12 (0.91, 1.00)
SVM	0.7333 ± 0.28 (0.49, 0.98)	0.8000 ± 0.30 (0.54, 1.00)	0.6667 ± 0.33 (0.37, 0.96)	0.7500 ± 0.28 (0.51, 0.99)	0.5778 ± 0.41 (0.22, 0.94)
Naive Bayes	1.0000 ± 0.00 (1.00, 1.00)	1.0000 ± 0.00 (1.00, 1.00)	1.0000 ± 0.00 (1.00, 1.00)	1.0000 ± 0.00 (1.00, 1.00)	1.0000 ± 0.00 (1.00, 1.00)

Best performing model is in bold text.

## Data Availability

The original contributions presented in this study are included in the article/Supplementary Material. Further inquiries can be directed to the corresponding author.

## Supplementary Materials

The following supporting information can be downloaded at: https://www.mdpi.com/article/10.3390/biom15070963/s1. Table S1: Description of Usher cell lines used in the mRNA biomarker study; Figure S1: Pathway analysis; Table S2: Pathway analysis performed using gProfiler() on the 58 mRNAs predicted by machine learning model. Pathway column shows the predicted pathways to be influenced by mRNAs of interest along with their gene ontology term id(GO), gene source, and *p*-value.
