# Supplementary material for: A Hybrid Sequential Feature Selection Approach for Identifying New Potential mRNA Biomarkers for Usher Syndrome Using Machine Learning
Source: Biomolecules. 2025 Jul 4;15(7):963. doi: 10.3390/biom15070963 (PMC12293090; doi:10.3390/biom15070963)
Supplement: Supplementary file 1 [file biomolecules-15-00963-s001.zip › biomolecules-3696359-supplementary.pdf]

Table S1. Description of Usher cell lines used in the mRNA biomarker study

Cell Line	D3739	D3741	D2880	Coriell cell line (GM09053)
Phenotype	Usher-1D	Usher-1B	Usher-3A	Usher-2A
Gene Name	CDH23	MYO7A	CLRN1	USH2A
Ref/Alt	G/A	G/T	A/C	AG/-<br>T/-
Chromosome position	10:71779316	11:77181589	3:150928107	1:216190280<br>1:215647526
Genotype	Homozygous	Homozygous	Homozygous	Heterozygous<br>Heterozygous
Inheritance	Recessive	Recessive	Recessive	Recessive<br>Recessive
Classification	Likely Pathogenic	Likely Pathogenic	VUS/Conflicting	Pathogenic<br>Pathogenic
Sequence ontology	Missense	Missense	Stop gained	Frameshift<br>Frameshift
ACMG Criteria	PM2, PS1, PP3	PM2, PP2, PS1, PP3	BS1, PVS1 Strong, PP5	PM2, PVS1, PP5<br>PM2, PVS1, PP5

Pathway Analysis

Pathway analysis was conducted using gprofiler to identify significantly enriched biological pathways associated with the given gene list. The analysis was performed in batches to optimize the processing of potentially large gene sets. Each batch of genes was analyzed for enrichment in various gene ontologies and pathways, including Gene Ontology (GO) categories (Biological Processes, Molecular Functions, Cellular Components), Kyoto Encyclopedia of Genes and Genomes (KEGG) pathways, Reactome (REAC) pathways, WikiPathways (WP), MSigDB, and PANTHER. A p-value threshold of 0.05 was applied to filter for statistically significant pathways. For each pathway returned by gprofiler, a list of genes that were involved in the pathway was extracted. The results, including pathway names, sources, p-values, and the list of genes, were compiled. Pathways with p-values below the significance threshold were considered significant for further analysis.

The pathway enrichment analysis shown in Fig S1 and Table S2, revealed several significant biological pathways that may be implicated in the underlying mechanisms of the studied condition. These pathways are categorized into developmental, neurotransmitter, epigenetic, transport, neuronal, and disease-related processes.

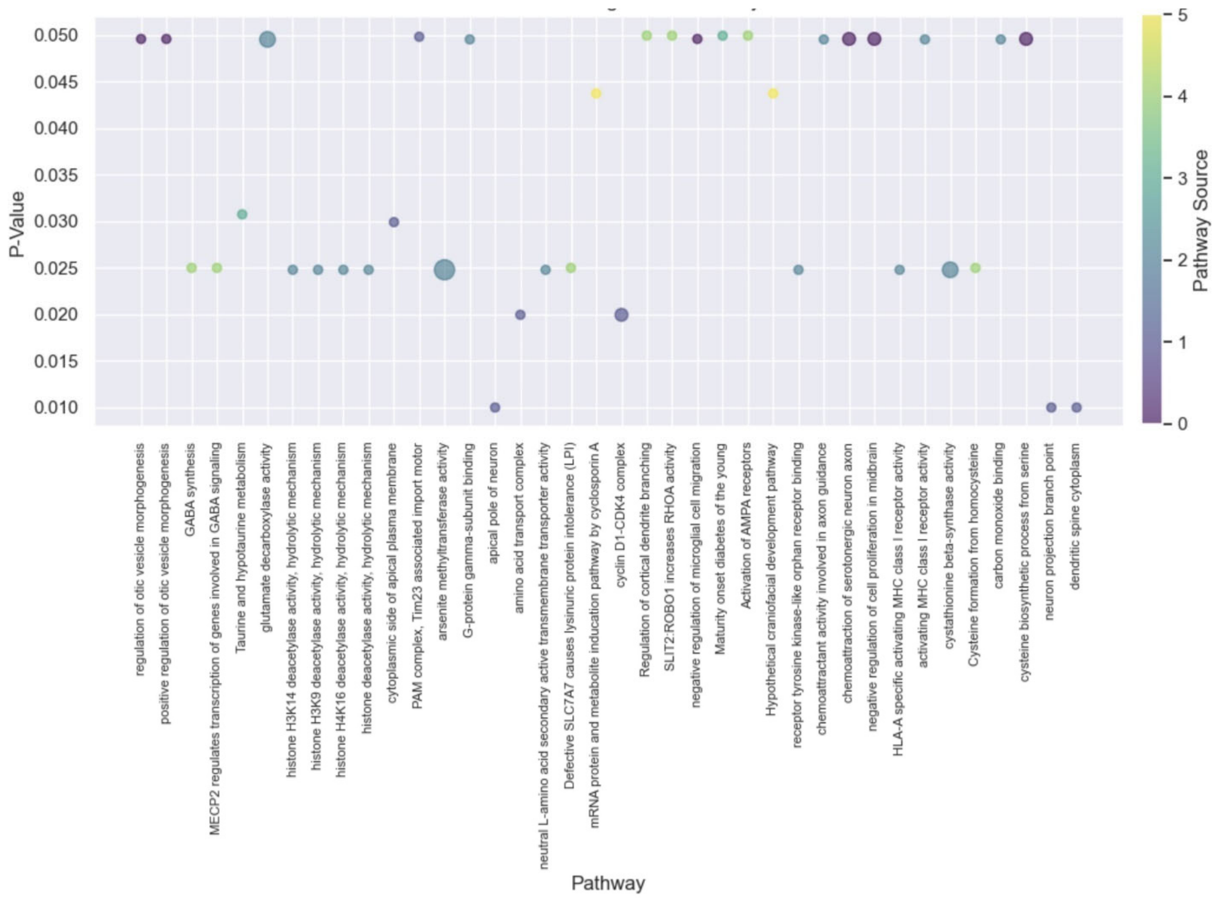

Figure S1. Pathway analysis.

Two pathways related to otic vesicle morphogenesis were identified: *regulation of otic vesicle morphogenesis* (p = 0.049563) and *positive regulation of otic vesicle morphogenesis* (p = 0.049563). These pathways are crucial for inner ear development, playing a key role in auditory function and congenital hearing disorders such as Usher syndrome [1,2,3]. Neurotransmitter-associated pathways, including GABA synthesis (p = 0.024961) and glutamate decarboxylase activity (p = 0.049515), were significantly enriched. Given that GABAergic and glutamatergic systems regulate neuronal excitability, these pathways are often implicated in neurodevelopmental disorders such as epilepsy and schizophrenia [4,5]. Additionally, taurine and hypotaurine metabolism (p = 0.030715) is associated with oxidative stress regulation and neuroprotection in various neurological diseases [6]. Pathways involved in transcriptional regulation and epigenetic modifications were identified, including *MECP2 regulation of transcription* (p = 0.024961) and several histone deacetylase (HDAC) activities (p = 0.024757). Mutations in MECP2 are well-documented in Rett syndrome, an X-linked neurodevelopmental disorder [7]. The involvement of histone deacetylases in gene silencing suggests potential epigenetic dysregulation contributing to neurodevelopmental and psychiatric disorders [8].

Table S2: Pathway analysis performed using gProfiler() on the 58 mRNAs predicted by machine learning model: Pathway column shows the predicted pathways to be influenced by mRNAs of interest along with their gene ontology term id(GO), gene source, and p-value.

Term ID	Pathway	Source	P-Value
GO:1904118	regulation of otic vesicle morphogenesis	GO:BP	0.04956
GO:1904120	positive regulation of otic vesicle morphogenesis	GO:BP	0.04956
REAC:R-HSA-888568	GABA synthesis	REAC	0.02496
REAC:R-HSA-9022927	MECP2 regulates transcription of genes involved in GABA signaling	REAC	0.02496
KEGG:00430	Taurine and hypotaurine metabolism	KEGG	0.03072
GO:0004351	glutamate decarboxylase activity	GO:MF	0.04951
GO:0031078	histone H3K14 deacetylase activity, hydrolytic mechanism	GO:MF	0.02476
GO:0032129	histone H3K9 deacetylase activity, hydrolytic mechanism	GO:MF	0.02476
GO:0034739	histone H4K16 deacetylase activity, hydrolytic mechanism	GO:MF	0.02476
GO:0141221	histone deacetylase activity, hydrolytic mechanism	GO:MF	0.02476
GO:0098592	cytoplasmic side of apical plasma membrane	GO:CC	0.02988
GO:0001405	PAM complex, Tim23 associated import motor	GO:CC	0.04980
GO:0030791	arsenite methyltransferase activity	GO:MF	0.02476
GO:0031682	G-protein gamma-subunit binding	GO:MF	0.04951
GO:0044225	apical pole of neuron	GO:CC	0.00996
GO:1990184	amino acid transport complex	GO:CC	0.01992
GO:0005294	neutral L-amino acid secondary active transmembrane transporter activity	GO:MF	0.02476
REAC:R-HSA-5660862	Defective SLC7A7 causes lysinuric protein intolerance (LPI)	REAC	0.02496
WP:WP3953	mRNA protein and metabolite inducation pathway by cyclosporin A	WP	0.04371
GO:0097128	cyclin D1-CDK4 complex	GO:CC	0.01992
REAC:R-HSA-8985801	Regulation of cortical dendrite branching	REAC	0.04992
REAC:R-HSA-8985586	SLIT2:ROBO1 increases RHOA activity	REAC	0.04992
GO:1904140	negative regulation of microglial cell migration	GO:BP	0.04956
KEGG:04950	Maturity onset diabetes of the young	KEGG	0.04991
REAC:R-HSA-399710	Activation of AMPA receptors	REAC	0.04992
WP:WP3655	Hypothetical craniofacial development pathway	WP	0.04371
GO:0005115	receptor tyrosine kinase-like orphan receptor binding	GO:MF	0.02476
GO:1902379	chemoattractant activity involved in axon guidance	GO:MF	0.04951
GO:0036517	chemoattraction of serotonergic neuron axon	GO:BP	0.04956
GO:1904934	negative regulation of cell proliferation in midbrain	GO:BP	0.04956
GO:0030108	HLA-A specific activating MHC class I receptor activity	GO:MF	0.02476
GO:0032397	activating MHC class I receptor activity	GO:MF	0.04951
GO:0004122	cystathionine beta-synthase activity	GO:MF	0.02476
REAC:R-HSA-1614603	Cysteine formation from homocysteine	REAC	0.02496
GO:0070025	carbon monoxide binding	GO:MF	0.04951
GO:0006535	cysteine biosynthetic process from serine	GO:BP	0.04956
GO:0061845	neuron projection branch point	GO:CC	0.00996
GO:0061846	dendritic spine cytoplasm	GO:CC	0.00996

Enriched pathways related to membrane transport and signaling included *amino acid transport complex* ($p = 0.019920$), *neutral L-amino acid secondary active transport* ($p = 0.024757$), and *G-protein gamma-subunit binding*

(p = 0.049515). Dysregulation in these pathways is often associated with cellular metabolism, neuronal communication, and metabolic disorders such as lysinuric protein intolerance. Neuronal function and synaptic plasticity pathways, such as *regulation of cortical dendrite branching* (p = 0.049923), *activation of AMPA receptors* (p = 0.049923), and *negative regulation of microglial cell migration* (p = 0.049563), were also enriched. These pathways are integral to synaptic transmission, learning, and memory, with implications in neurodegenerative diseases such as Alzheimer's disease and autism spectrum disorders [8,9]. Several pathways were directly associated with disease mechanisms, including *maturity onset diabetes of the young* (p = 0.049912), *HLA-A specific activating MHC class I receptor activity* (p = 0.024757), and *cysteine biosynthesis from homocysteine* (p = 0.024961). These pathways suggest metabolic, immune, and inflammatory involvement, linking neurological and systemic disorders [10,11].
The pathway enrichment analysis highlights key biological mechanisms underlying the studied condition. The identification of pathways related to otic development, neurotransmission, epigenetic regulation, and metabolic processes suggests potential biomarkers and therapeutic targets. Future studies should focus on functional validation of these pathways to elucidate their precise roles in disease pathogenesis.

References

1. Kelley, M. W. (2006). Regulation of cell fate in the sensory epithelia of the inner ear. Nature Reviews Neuroscience, 7(11), 837-849.
2. Petit, C., & Richardson, G. P. (2009). Linking genes underlying deafness to hair-bundle development and function. Nature neuroscience, 12(6), 703-710.
3. Treiman, D. M. (2001). GABAergic mechanisms in epilepsy. Epilepsia, 42, 8-12.
4. Coyle, J. T. (2006). Glutamate and schizophrenia: beyond the dopamine hypothesis. Cellular and molecular neurobiology, 26, 363-382.
5. Ripps, H., & Shen, W. (2012). taurine: a "very essential" amino acid. Molecular vision, 18, 2673.
6. Amir, R. E., Van den Veyver, I. B., Wan, M., Tran, C. Q., Francke, U., & Zoghbi, H. Y. (1999). Rett syndrome is caused by mutations in X-linked MECP2, encoding methyl-CpG-binding protein 2. Nature genetics, 23(2), 185-188.
7. Abel, T., & Zukin, R. S. (2008). Epigenetic targets of HDAC inhibition in neurodegenerative and psychiatric disorders. Current opinion in pharmacology, 8(1), 57-64.
8. Shepherd, J. D., & Huganir, R. L. (2007). The cell biology of synaptic plasticity: AMPA receptor trafficking. Annu. Rev. Cell Dev. Biol., 23(1), 613-643.
9. Hong, S., Beja-Glasser, V. F., Nfonoyim, B. M., Frouin, A., Li, S., Ramakrishnan, S., ... & Stevens, B. (2016). Complement and microglia mediate early synapse loss in Alzheimer mouse models. Science, 352(6286), 712-716.
10. Fajans, S. S., & Bell, G. I. (2011). MODY: history, genetics, pathophysiology, and clinical decision making. Diabetes care, 34(8), 1878-1884.
11. Klein, J. A. N., & Sato, A. (2000). The HLA system. New England journal of medicine, 343(10), 702-709.
